# Chemical Composition of Volatile Compounds in *Apis mellifera* Propolis from the Northeast Region of Pará State, Brazil

**DOI:** 10.3390/molecules26113462

**Published:** 2021-06-07

**Authors:** Mozaniel Santana de Oliveira, Jorddy Neves Cruz, Oberdan Oliveira Ferreira, Daniel Santiago Pereira, Natanael Santiago Pereira, Marcos Enê Chaves Oliveira, Giorgio Cristino Venturieri, Giselle Maria Skelding Pinheiro Guilhon, Antônio Pedro da Silva Souza Filho, Eloisa Helena de Aguiar Andrade

**Affiliations:** 1 Programa de Pós-Graduação em Biodiversidade e Biotecnologia—Rede Bionorte, Universidade Federal do Pará, Rua Augusto Corrêa S/N, Guamá, Belém 66075-900, PA, Brazil; jorddynevescruz@gmail.com (J.N.C.); oberdan@museu-goeldi.br (O.O.F.); eloisa@museu-goeldi.br (E.H.d.A.A.); 2 Laboratório Adolpho Ducke-Coordenação de Botânica, Museu Paraense Emílio Goeldi, Av. Perimetral, 1901, Terra Firme, Belém 66077-830, PA, Brazil; 3 Empresa Brasileira de Pesquisa Agropecuária-Embrapa Amazônia Oriental, Tv. Dr. Eneas Pinheiro, s/n—Marco, Belém 66095-903, PA, Brazil; daniel.pereira@embrapa.br (D.S.P.); marcos-ene.oliveira@embrapa.br (M.E.C.O.); antonio-pedro.filho@embrapa.br (A.P.d.S.S.F.); 4 Laboratory of Soil Water for Irrigation Purposes and Vegetable Tissues, Federal Institute of Education Science and Technology of Ceará, Limoeiro do Norte 62930-000, CE, Brazil; natanaelsan@hotmail.com; 5 Pollination Ecology, Meliponiculture and Beekeeping, NATIVO Company, Wavell Heights North, QLD 4012, Australia; giorgio@venturieri.com; 6 Faculdade de Química, Universidade Federal do Pará, Rua Augusto Corrêa S/N, Guamá, Belém 66075-900, PA, Brazil; giselle@ufpa.br

**Keywords:** Amazon, bioproducts, propolis, aroma, bioactive compounds

## Abstract

Propolis is a balsamic product obtained from vegetable resins by exotic Africanized bees *Apis mellifera* L., transported and processed by them, originating from the activity that explores and maintains these individuals. Because of its vegetable and natural origins, propolis is a complex mixture of different compound classes; among them are the volatile compounds present in the aroma. In this sense, in the present study we evaluated the volatile fraction of propolis present in the aroma obtained by distillation and simultaneous extraction, and its chemical composition was determined using coupled gas chromatography, mass spectrometry, and flame ionization detection. The majority of compounds were sesquiterpene and hydrocarbons, comprising 8.2–22.19% α-copaene and 6.2–21.7% β-caryophyllene, with additional compounds identified in greater concentrations. Multivariate analysis showed that samples collected from one region may have different chemical compositions, which may be related to the location of the resin’s production. This may be related to other bee products.

## 1. Introduction

Honeybees are among the most studied insects because of their high economic value and fundamental role in agriculture and ecosystems [1]. The species *Apis mellifera* is known worldwide as an important pollinator of agricultural crops [2]. This species is native to Europe, Africa, the Middle East, and parts of Asia [3,4], and has great potential for adaptation to different biomes and climatic conditions [3]. *Apis mellifera* is not restricted to honey production; it also produces propolis through the addition of saliva and wax to organic liquids collected from plant sap, resin, gum, and latex [5].

Propolis, also called “bee glue”, is a resinous substance similar in some aspects to natural wax found in hives [6]. This substance has a dark yellow to brown color and is formed from materials collected by bees from flower buds, leaves, and other plant parts [7]. Propolis is sticky and adhesive in nature [8]. For bees, propolis is of paramount importance, as the insects use it as a coating to seal cracks or spaces in the hive, a base for making honey [6], colony protection, and defense against infections and parasites [9].

The protection that propolis offers to bees is related to the pharmacological properties of this bioactive product [10], as indicated by its uses in traditional medicine. Propolis is mainly used for treating diseases of the vascular and blood system (anemia), respiratory infections, ulcers, mycoses, and cancer, along with improving the immune system [11]. The chemical characteristics of propolis are directly related to their biological activity [12,13]. Previous literature has discussed the great potential of propolis as an antimicrobial and antioxidant material [14,15] with immunological, antiparasitic, and cytotoxic properties [16], as well as antiviral activity against the SARS-Cov-2 virus [17].

Several products based on propolis, mainly as drinks and health foods, have been commercialized. The function of propolis as a supplement and bioactive food preservative has caused constant growth in the demand for similar products [9,18]. Users of this bee product have gained great benefits related to the biological activities and volatile components of propolis [8].

Propolis is a phytochemical and complex mixture composed of 50% resin (containing flavonoids and 66 phenolic acids), 30% wax, 10% essential oil, 5% pollen, and 5% other organic compounds [5,19]. Studies have reported that the volatile compound profile of propolis comprises a variety of chemicals and volatile compounds such as 1-methyl-naphthalene, naphthalene, 3-methyl-1-butanol [20], limonene, *β*-caryophyllene, nerolidol [21], (*E*)-isoeugenol, linalool, butanoic acid [22], and acetophenone [23]. The chemical composition of propolis is related to the biosynthetic capacity of plants and their secondary metabolites used by bees [9,24]. In this context, the present study aims to evaluate the chemical composition of the volatile fraction of seven samples of propolis collected in the Northeast of Pará, Eastern Amazon.

## 2. Results and Discussion

The chemical composition of the volatile compounds of the different propolis samples from *Apis mellifera*, as analyzed by gas chromatography mass spectroscopy (GC-MS) and gas chromatography flame ionization detection (GC-FID) are shown in Table 1. In total, 87 compounds were identified, demonstrating the diverse chemical composition of the volatile compounds in the propolis. Chi et al. [25] identified approximately 406 compounds, mainly comprising monoterpenes, phenol alcohols, sesquiterpenoids, acid esters, aldoketones, and hydrocarbons. However, in the present study, the predominant classes were sesquiterpene hydrocarbons (80.6–89.2%), followed by oxygenated monoterpenes (3.6–8.4%). The class of phenylpropanoids (0.2%), was identified only in sample 3 (Table 1). Phenyl propanoids are the predominant class in red propolis from Brazil, followed by hydrocarbon sesquiterpenes [16]. In propolis from the Cerrado biome (Campo Grande, MS, Brazil), sesquiterpenes, hydrocarbons, and oxygenated compounds are the main components [26].

**Figure 1:**
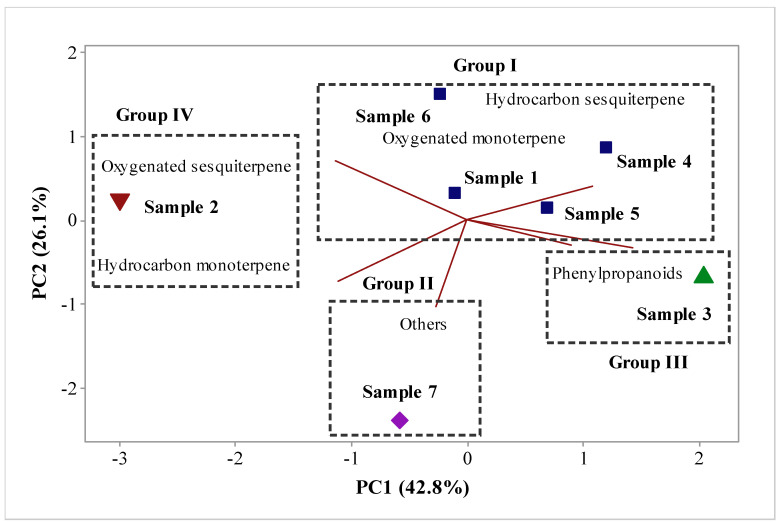
Biplot (principal component analysis) from the analysis of volatile compound classes identified in the aromas of seven samples of bee propolis from *Apis mellifera*.

**Figure 2:**
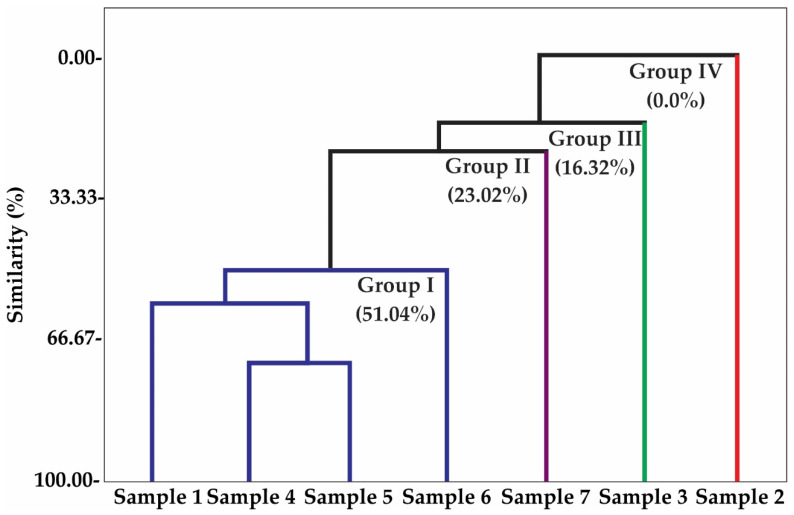
Dendrogram presenting the relational similarity of the volatile compound classes identified in the aromas of seven bee propolis samples from *Apis mellifera*.

Multivariate analyses, principal component analysis (PCA) (Figure 1), and hierarchical cluster analysis (HCA) were performed to analyze the correlation between the classes of compounds identified in the different samples, as shown in Figure 1 and Figure 2. As shown in Figure 1, the principal components (PC) contained the main components analyzed, PC1 and PC2, which accounted for 42.8% and 26.1% of the variables, respectively. In combination, both variables accounted for 68.9% of the variance in the analyzed data. In the HCA analysis, the similarity between the identified classes was evaluated; four groups were observed. Group I, including samples 1, 4, 5, and 6, showed a similarity of 51.04% (Figure 2), and comprised oxygenated monoterpenes and hydrocarbon sesquiterpenes (Figure 1). Groups II, III, and IV contained only one sample each and comprised phenylpropanoids, oxygenated sesquiterpenes, and hydrocarbon monoterpenes, with similarities of 23.02%, 16.32%, and 0%, respectively (Figure 2). Because of the complex chemical composition of propolis, chemometric analysis is widely used in studies to define groups of chemically correlated samples [27,28,29,30,31,32].

The compounds identified at the highest concentrations are listed in Table 1. Sample 1 contained the following: naphthalene (4.3%), α-copaene (8.2%), cyperene (12.6%), β-caryophyllene (11.8%), (*E*)-β-farnesene (6.6%), β-selinene (9.9%), α-selinene (9.8%), and δ-cadinene (5.4%). The composition of sample 2 is as follows: α-copaene (15.4%), (*Z*)-α-bergamotene (5.3%), β-caryophyllene (9%), (*E*)-α-bergamotene (22.1%), and δ-cadinene (4.4%). For sample 3, the components present were: (*E*)-anethole (5.3%), α-copaene (17.1%), (*Z*)-α-bergamotene (4.5%), β-caryophyllene (6.2%), (*E*)-α-bergamotene (19.1%), β-bisabolene (7%), and δ-cadinene (5.9%). Sample 4 contained the following: naphthalene (7.4%), α-copaene (16.2%), β-caryophyllene (7.9%), (*E*)-α-bergamotene (4.9%), (*E*)-β-farnesene (7%), γ-muurolene (6.6%), β-bisabolene (4%), and δ-cadinene (12.6%). In sample 5, naphthalene (5.8%), α-copaene (9.4%), cyperene (10%), β-caryophyllene (21.7%), (*E*)-β-farnesene (8%), β-bisabolene (7.8%), and δ-cadinene (4.3%). Sample 6 contained naphthalene (5%), α-copaene (14.3%), cyperene (4.6%), β-caryophyllene (13.6%), (*E*)-α-bergamotene (7.1%), β-bisabolene (10%), δ-cadinene (7%), β-bisabolene (10%), and δ-cadinene (7%). For sample 7, naphthalene (4.99%), α-cubebene (5.01%), α-copaene (22.19%), α-gurjunene (7.77%), β-caryophyllene (17.69%), α-humulene (4.3%), and β-bisabolene (5.46%) were present. These results are qualitatively similar to those reported in the literature [19,33].

In other studies, the major compounds were thymol (29.61%), its isomer carvacrol (30.57%) from Kermanshah City in the west of Iran [34], carvone (40.34%), β-bisabolene (10.6%), β-thujone (11.45%), carvone (40.34%) from Tehran Province, Iran [35], carvacrol (20.7%), acetophenone (13.5%), spathulenol (11.0%), (*E*)-nerolidol (9.7%), β-caryophyllene (6.2%) from Atlantic Forest in São Lourenço MG, Brazil [36], β-pinene (2.0–21.8%), α-pinene (1.2–46.5%), limonene (11.6%), dihydrosabinene (17.8%), **1**,**8**-cineole (0.1–11.0%), *p*-cymene (0.1–5.3%), **2**,**7**-dimethyl-**3**-octen-**5**-yne (trace-11.7%), octanal (12.9%), (*E*)-β-ocimene (17.8%), α-thujene (trace-11.0%), and styrene (13.5%) from South Africa [37], δ-cadinene (1.29–13.31%), γ-cadinene (1.36–8.85%) and α-muurolene (0.78–6.59%), β-eudesmol (2.33–12.83%), T-cadinol (2.73–9.95%) and α-cadinol (4.84–9.74%) from different Italian regions [19], and α-pinene, β-pinene, γ-terpinene, α-muurolene, γ-cadinene and δ-cadinene from different regions of Croatia [38].

**Figure 3:**
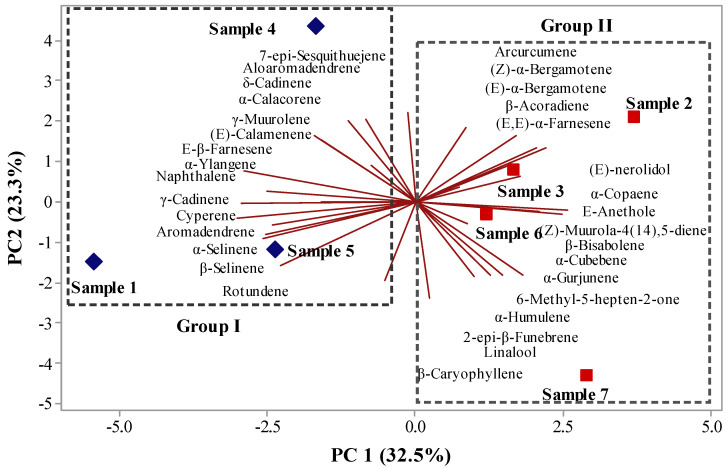
Biplot (PCA) from the analysis of volatile compounds identified in the aromas of seven samples of bee propolis from *Apis mellifera*.

**Figure 4:**
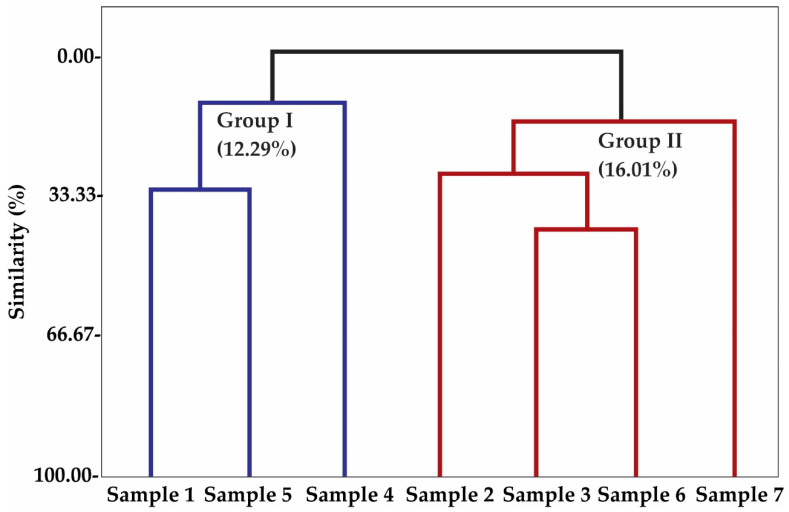
Dendrogram representing the relational similarity of the volatile compounds identified in the aromas of seven bee propolis samples from *Apis mellifera*.

Multivariate analysis, principal component analysis (PCA) (Figure 3), and hierarchical cluster analysis (HCA) (Figure 4) were applied to the chemical compounds identified in the different volatile compounds present in the aroma fractions of propolis samples from *Apis mellifera*. The first component PC1 accounted for 32.5% of the variation, while PC2 accounted for 23.3% of the variation. Combined, both components comprised 55.8% of the variance (Figure 3). HCA, considering the Euclidean distances and complete bonds, confirmed the formation of two distinct groups, without group I shown in Figure 1. The first of these, formed by samples I, IV, V, and VI, with a similarity of 12.29% (Figure 4), comprised **7**-epi-sesquithuejene, allo-aromadendrene, δ-cadinene, sesquicineole, (*E*)-calamenene, β-sesquiphelandrene, (*E*)-cadina-**1**,**4**-diene, α-cadinene, α-calacorene, γ-muurolene, (*E*)-calamenene, (E)-β-farnesene, α-langene, naphthalene, γ-cadinene, cyperene, aromadendrene, α-selimene, β-selimene, and rutundene (Figure 3). The second group was formed by grouping samples II, III, VI, and VII, with a similarity of 16.01% (Figure 4). This arose from the consolidation of the following compounds: arcucumene, (*Z*)-α-bergamotene, β-acoradiene, (*E*,*E*)-α-farmasene, (*E*)-nerolidol, α-copaene, (*E*)-anthole, (*Z*)-muurola-**4**(**14**),**5**-diene, β-bisabolene, α-cubebene, **6**-methyl-**5**-hepten-**2**-one, α-humulene, **2**-epi-b-funebrene, linalool, and β-carophyllene (Figure 3).

The difference between the chemical composition of the present samples (Table 1) and those reported in the literature may be related to the geographical origin and the biome in which the bees collected the raw materials to form the propolis [8]. The isolation and analysis techniques [39] can also directly influence the chemical composition of both the volatile compounds and compounds of higher molecular weight, or those with greater polarity [40]. Olegário et al. [41] used PCA to determine the volatile compounds that quantitatively constituted propolis samples collected in different regions of Brazil. The geographic origin of the samples influenced their chemical composition in all the cases analyzed by the authors.

Because propolis is a product of plant origin, its chemical composition depends on factors including local flora, place of collection, and the seasonal and circadian period of collection of raw materials by bees, as the plants producing volatile compounds tend to produce different compounds at different times. The period of the year, climate and temperature, and rainfall index, among other factors, can induce variability in the chemical composition of propolis. Furthermore, the volatile compounds identified in propolis can be added to other analyses of chemical composition and serve as markers to identify their botanical origin [8,42]. This was also observed in propolis samples from Morocco [43], the northeastern states of Brazil [44], Yemen [45], other regions of Brazil, Estonia, China, Uruguay [46], South Africa [37], and Argentina [21]. Volatile compounds constitute a small fraction of propolis and are important for characterizing its botanical origin [47]. In addition, volatile compounds can be used as food preservatives in propolis-based packaging [48] by exploiting their antioxidant [15,25], antifungal [49,50], antibacterial [51], and other biological activities [23].

**Table 1:** Chemical composition (%) of volatile compounds identified in different propolis samples of *Apis mellifera* collected in the city of São João de Pirabas state of Pará.

Constituent	RI_L_	RI_C_	Sample 1	Sample 2	Sample 3	Sample 4	Sample 5	Sample 6	Sample 7
**2**-Heptanone	889 ^a^	888		0.4					0.45
α-Pinene	932 ^a^	933	0.2	0.8	0.6	0.4	0.5	0.7	0.86
Benzaldehyde	952 ^a^	953	0.1	1					0.61
**6**-Methyl-**5**-hepten-**2**-one	981 ^a^	985	0.5	0.7	0.7	0.5	1.2	0.8	1.16
*p*-Cymene	1020 ^a^	1022	0.1	0.1	0.1	0.1		0.1	0.1
Limonene	1024 ^a^	1025	0.1	0.1	0.1	0.1		0.1	0.1
**1**,**8**-Cineole	1026 ^a^	1027		0.2		0.1		0.1	0.1
(*Z*)-Linalool oxide (furanoid)	1067 ^a^	1069	0.1	0.4	0.1	0.1		0.1	0.2
*trans*-Linalool oxide (furanoid)	1084 ^a^	1090	0.1	0.1	0.1	0.1		0.1	0.33
Linalool	1095 ^a^	1100	0.8	0.6	0.8	0.5	1.3	0.7	1.51
Naphthalene	1178 ^a^	1182	4.3	1.4	1.1	7.4	5.8	5	4.99
Methyl chavicol	1195 ^a^	1197	0.3	0.2	0.8	0.3	0.4	0.3	0.3
β-Cyclocitral	1217 ^a^	1217			0.1				
*n*-Decanal	1201 ^a^	1229		0.3					0.06
Neral	1235 ^a^	1235		0.1					
Geranial	1264 ^a^	1266		0.1					0.1
Benzenepropanoic acid. methyl ester	1278 ^b^	1272							0.09
(*Z*)-Methyl cinnamate	1299 ^a^	1280		0.1					
(*E*)-Anethole	1282 ^a^	1282			5.3				
**2**-Undecanone	1293 ^a^	1292							0.17
Tridecane	1300 ^a^	1300							0.1
α-Cubebene	1345 ^a^	1345	1.3	2.6	3.7	3.4	1.1	2.1	5.01
α-Ylangene	1373 ^a^	1367	1.6	0.9	0.9	1.3	1.5	1.6	0.7
α-Copaene	1374 ^a^	1375	8.2	15.4	17.1	16.2	9.4	14.3	22.19
β-Patchoulene	1379 ^a^	1378	0.5				0.2		
**2**-epi-α-Funebrene	1380 ^a^	1380		0.3				0.6	0.26
α-Duprezianene	1387 ^a^	1387				0.3			
β-Bourbonene	1387 ^a^	1387	0.3		0.1		0.3		
β-Elemene	1389 ^a^	1389	0.5						
**7**-epi-Sesquithuejene	1390 ^a^	1391		0.7		1.5	0.5	0.6	
Cyperene	1398 ^a^	1398	12.6	1.1	3.2	3.6	10	4.6	
α-Gurjunene	1409 ^a^	1400	0.8	0.7	0.5	0.7		0.5	7.77
(*Z*)-α-Bergamotene	1411 ^a^	1411		5.3	4.5	2	2.5	2.3	
**2**-epi-β-Funebrene	1411 ^a^	1412						2	3.3
β-Caryophyllene	1417 ^a^	1418	11.8	9	6.2	7.9	21.7	13.6	17.69
β-Cedrene	1419 ^a^	1421		0.4		0.4		0.5	0.25
β-Copaene	1430 ^a^	1426	0.3	0.1	0.3	0.4	0.4		0.26
(*E*)-α-Bergamotene	1432 ^a^	1430	1.4	22.1	19.1	4.9	2.8	7.1	1.81
α-Guaiene	1437 ^a^	1434							0.31
**6**,**9**-Guaiadiene	1442 ^a^	1437	0.6	0.4	0.7	0.6		0.4	0.13
Aromadendrene	1439 ^a^	1439	1.7		0.1	0.1		0.2	
*trans*-Muurola-**3**,**5**-diene	1451 ^a^	1445							0.88
α-Humulene	1452 ^a^	1451		0.9	0.5	0.1	1	3	4.3
(*E*)-β-Farnesene	1454 ^a^	1454	6.6	1	2.8	7	8	3	
Rotundene	1457 ^a^	1456	2		0.7	0.1	1	0.1	0.83
Allo-aromadendrene	1458 ^a^	1458	0.5	0.6		1.7	0.4		0.07
(*Z*)-cadina-**1**(**6**),**4**-diene	1461 ^a^	1467		0.8	0.9	0.9			0.68
(*Z*)-Muurola-**4**(**14**),**5**-diene	1465 ^a^	1470		1.7	1.9			2.9	1.49
**4**,**5**-di-epi-Aristolechene	1471 ^a^	1471	0.5						
β-Acoradiene	1469 ^a^	1474		0.9	0.8	1	0.7	1	0.7
γ-Gurjunene	1475 ^a^	1475	0.5						
ar-Curcumene	1479 ^a^	1477	0.4	1	1	1.5	0.7	1.7	0.49
γ-Muurolene	1478 ^a^	1478	1			6.6	3.3		
β-Selinene	1489 ^a^	1483	9.9	0.6	0.7	1	2.2	1.6	1.53
(*E*)-Muurola-**4**-(**14**),**5**-diene and	1493 ^a^	1486		0.5	0.8	0.7	0.4	0.3	0.48
α-Selinene	1498 ^a^	1489	9.8	1.3	1.3	1.5	2.3	2.3	1.64
α-Muurolene	1500 ^a^	1493		0.3	0.6	0.9	0.4	0.7	0.45
Cis-cadina-**1**,**4**-diene	1495 ^a^	1495						0.8	
(*E*)-β-guaiene	1502 ^a^	1497		0.2			0.4	0.3	0.19
β-Bisabolene	1505 ^a^	1504	2.3	3.4	7	4	7.8	10	5.46
(*E,E*)-α-Farnesene	1505 ^a^	1505		1.1					
γ-Cadinene	1513 ^a^	1507	1.2	0.1	0.7	1.1	1.3	0.8	0.48
δ-Cadinene	1522 ^a^	1513	5.4	4.4	5.9	12.6	4.3	7	3.76
Sesquicineole	1515 ^a^	1515		0.1					
(*E*)-Calamenene	1521 ^a^	1516	1.9	1.1	2.3	2.4	1.2	1.9	1.64
β-Sesquiphelandrene	1521 ^a^	1521		0.5					
(*E*)-cadina-**1**,**4**-diene	1533 ^a^	1527	0.3	0.4	0.3	0.5		0.3	0.25
α-Cadinene	1537 ^a^	1530	0.4	0.1	0.2	0.3	0.6	0.2	0.12
α-Calacorene	1544 ^a^	1535	0.6	0.4	0.6	1	0.6	0.1	0.28
β-Calacorene	1564 ^a^	1544						0.6	
Elemicin	1555 ^a^	1555			0.2				
(*E*)-Nerolidol	1561 ^a^	1558	0.2	1.2		0.3	0.1	0.3	0.43
Caryophyllenyl alcohol	1570 ^a^	1570						0.1	
Caryolan-**8**-ol	1571 ^a^	1571							0.1
Caryophyllene oxide	1582 ^a^	1576	0.4	0.1	0.1	0.2	0.5	0.3	0.2
Sphatulenol	1577 ^a^	1577	0.1						
Gleenol	1586 ^a^	1590			0.1			0.1	
Hexadecane	1600 ^a^	1600	0.1						0.1
Junenol	1618 ^a^	1603	0.2	0.3	0.1	0.3	0.3	0.6	0.18
α-Corocalene	1622 ^a^	1622			0.1	0.1		0.2	
**1**,**10**-di-epi-Cubenol	1618 ^a^	1623	0.2	0.2	0.2	0.3	0.1	0.3	0.17
Cubenol	1514 ^a^	1638	0.2	0.2	0.2	0.3		0.4	0.19
α-Cadinol	1652 ^a^	1650	0.2	0.1	0.1	0.1	0.1	0.2	0.11
Cadalene	1675 ^a^	1667		0.2					0.09
β-Bisabolol	1674 ^a^	1674		0.4	0.1		0.1		
epi-α-Bisabolol	1683 ^a^	1683	0.1		0.1	0.2	0.1	0.2	
α-Bisabolol	1685 ^a^	1685				0.1		0.1	0.12
Hydrocarbon monoterpene			0.9	3	1.4	1	1.7	1.6	3.18
Oxygenated monoterpene			5.7	3.6	8.4	8.6	7.5	6.4	7.78
Hydrocarbon sesquiterpene			84.9	80.6	85.5	88.3	87	89.2	85.49
Oxygenated sesquiterpene			1.6	2.5	1	1.8	1.3	2.6	1.5
Phenylpropanoids					0.2				
Others			0.1						0.37
Total			93.2	89.7	96.5	99.7	97.5	99.8	98.32

Org = organic; Min = mineral; Cont. = control; RI(C): Calculated Retention Index; RI(L): Literature Retention Index. (a) Adams [52]; and (b) Nist [53].

## 3. Materials and Methods

### 3.1. Collection Area

*Apis mellifera* propolis samples were collected in apiaries located in the city of São João de Pirabas, which is in the northeastern region of the state of Pará-Eastern Amazon (geographic coordinates: 0°46′08″ S 47°10′26″ O). The samples were collected from seven different hives from a producer. The hives were arranged at a distance of 2 m from each other in a forest with different types of plants, as shown in the Supplementary Material S1. Propolis was collected with the aid of sterile spatulas. According to the methodology described by Dutra et al. [54], the samples were placed in sterile plastic bags and kept at a temperature of 5–10 °C after collection, see Supplementary Material S1.

### 3.2. Aroma Extraction

Before the aroma extraction process, the propolis samples were frozen and crushed. For aroma extraction, 10 g of the sample was mixed with water (20 mL) and subjected to simultaneous distillation–extraction (SDE) for 3 h using a Chrompack Micro-Steam Distillation Extractor (Likens–Nickerson) and pentane (2 mL) as the organic mobile phase, as described in the literature [55,56].

### 3.3. Analysis of Chemical Composition of Volatile Compounds

The chemical compositions of the volatile fraction of the seven propolis samples was analyzed using GC-MS via a Thermo DSQ-II system equipped with a DB-5MS silica capillary column (30 m × 0.25 mm; 0.25 mm). For this analysis, the following conditions were used: the temperature was increased from 60 to 240 °C at a rate of 3 °C/min; the injector temperature was set to 240 °C; helium was used as the carrier gas (linear velocity of 32 cm/s, measured at 100 °C); aqueous 2:1000 *n*-hexane was injected in one step (0.1 mL); the temperature of the ion source and other parts was set at 200 °C. The quadrupole filter was swept in the range of 39–500 Da every second. Ionization was achieved by using an electronic impact technique at 70 eV. The volatile components were identified by comparison with the literature [52,53]. The volatile constituents were quantified by peak-area normalization using the FOCUS GC/FID, as previously reported by our research group [42].

### 3.4. Statistical Analysis

Multivariate analysis was performed according to a previously reported methodology [42,57,58] using Minitab 17^®^ software (free version, Minitab Inc., State College, PA, USA). The chemical constituents of the essential oils were used as the variables. The raw data were first standardized to the same “weight.” PCA was then performed using the matrix type correlation configuration in the software. In the HCA of the samples, the Euclidean distance options were used for distance measurement, and the connection method used was complete. Multivariate analysis was applied to the samples, where the concentration of the compounds was ≥1%.

## 4. Conclusions

Different volatile compounds present in the aroma were obtained from the analyzed samples of propolis. Compounds belonging to the sesquiterpene class were present in the highest concentrations. Variability of the samples was observed using multivariate analysis. This may be related to the bee collection area. Based on the analyzed data, different groups were delineated, both for the classes of compounds and for the compounds analyzed in the form of a correlation matrix. These data are important because they can provide guidelines for future studies on the botanical origins of propolis.

## Data Availability

Not applicable.

## Supplementary Materials

The following are available online, Figure S1: Collection of propolis samples.

## Sample Availability

Samples of the compounds in the Museu Paraense Emílio Goeldi are available from the authors.
