# Supplementary material for: Chemical Composition of Volatile Compounds in Apis mellifera Propolis from the Northeast Region of Pará State, Brazil
Source: Molecules. 2021 Jun 7;26(11):3462. doi: 10.3390/molecules26113462 (PMC8201256; doi:10.3390/molecules26113462)

Supplementary material S1

Apis Mellifera bee propolis sample collection site used in the present study

Chemical Composition of Volatile Compounds in *Apis mellifera* Propolis from the Northeast Region of Pará State, Brazil

Mozaniel Santana de Oliveira [1,2,*], Jorddy Neves Cruz [1], Oberdan Oliveira Ferreira [1], Daniel Santiago Pereira [3], Natanael Santiago Pereira [4], Marcos Enê Chaves Oliveira [3], Giorgio Cristino Venturieri [5], Giselle Maria Skelding Pinheiro Guilhon [6], Antônio Pedro da Silva Souza Filho [3] and Eloisa Helena de Aguiar Andrade [1,2,6]

1 Programa de Pós-Graduação em Biodiversidade e Biotecnologia–Rede Bionorte, Universidade Federal do Pará, Rua Augusto Corrêa S/N, Guamá, Belém 66075-900, PA, Brazil; jorddynevescruz@gmail.com (J.N.C.); oberdan@museu-goeldi.br (O.O.F.); eloisa@museu-goeldi.br (E.H.d.A.A.)
2 Laboratório Adolpho Ducke-Coordenação de Botânica, Museu Paraense Emílio Goeldi, Av. Perimetral, 1901, Terra Firme, Belém 66077-830, PA, Brazil
3 Empresa Brasileira de Pesquisa Agropecuária-Embrapa Amazônia Oriental, Tv. Dr. Eneas Pinheiro, s/n - Marco, Belém, PA 66095-903, Brazil; daniel.pereira@embrapa.br (D.S.P.); marcos-ene.oliveira@embrapa.br (M.E.C.O.); antonio-pedro.filho@embrapa.br (A.P.d.S.S.F.)
4 Laboratory of soil water for irrigation purposes and vegetable tissues, Federal Institute of Education Science and Technology of Ceará, Limoeiro do Norte, 62930-000, CE, Brazil; natanaelsan@hotmail.com
5 Pollination Ecology, Meliponiculture and Beekeeping, NATIVO Company, Wavell Heights North, 4012, QLD, Australia; giorgio@venturieri.com
6 Faculdade de Química, Universidade Federal do Pará, Rua Augusto Corrêa S/N, Guamá, Belém 66075-900, PA, Brazil; giselle@ufpa.br
* Correspondence: mozanieloliveira@museu-goeldi.br; Tel.: +55-91-988647823

Citation: de Oliveira, M.; Cruz, J.N.; Ferreira, O.O.; Pereira, D.S.; Pereira, N.S.; Oliveira, M.E.C.; Venturieri, G.C.; Guilhon, G.M.S.P.; da Silva Souza Filho, A.; de Aguiar Andrade, E. Chemical Composition of Volatile Compounds in *Apis mellifera* Propolis from the Northeast Region of Pará State, Brazil. *Molecules* **2021**, *26*, x. https://doi.org/10.3390/xxxxx

Academic Editor: Juraj Majtan

Received: 13 April 2021
Accepted: 20 May 2021
Published: date

Publisher's Note: MDPI stays neutral with regard to jurisdictional claims in published maps and institutional affiliations.

Copyright: © 2021 by the authors. Submitted for possible open access publication under the terms and conditions of the Creative Commons Attribution (CC BY) license (http://creativecommons.org/licenses/by/4.0/).

Abstract: Propolis is a balsamic product obtained from vegetable resins by exotic Africanized bees *Apis mellifera* L., transported and processed by them, originating from the activity that explores and maintains these individuals. Because of its vegetable and natural origins, propolis is a complex mixture of different compound classes; among them are the volatile compounds present in the aroma. In this sense, in the present study we evaluated the volatile fraction of propolis present in the aroma obtained by distillation and simultaneous extraction, and its chemical composition was determined using coupled gas chromatography, mass spectrometry, and flame ionization detection. The majority of compounds were sesquiterpene and hydrocarbons, comprising 8.2%–22.19% α-copaene and 6.2%–21.7% β-caryophyllene, with additional compounds identified in greater concentrations. Multivariate analysis showed that samples collected from one region may have different chemical compositions, which may be related to the location of the resin's production. This may be related to other bee products.

Keywords: Amazon; bioproducts; propolis; aroma; bioactive compounds

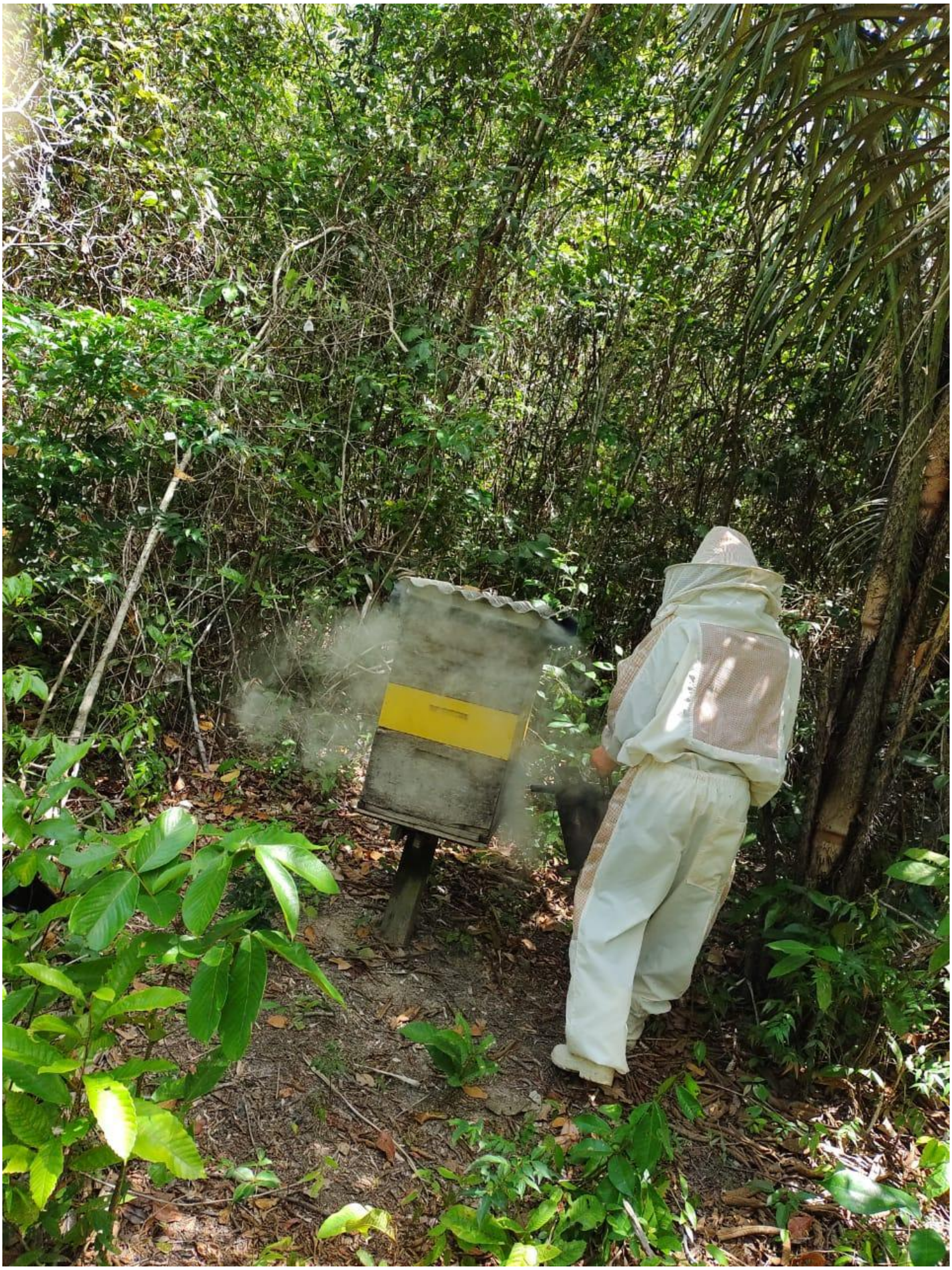

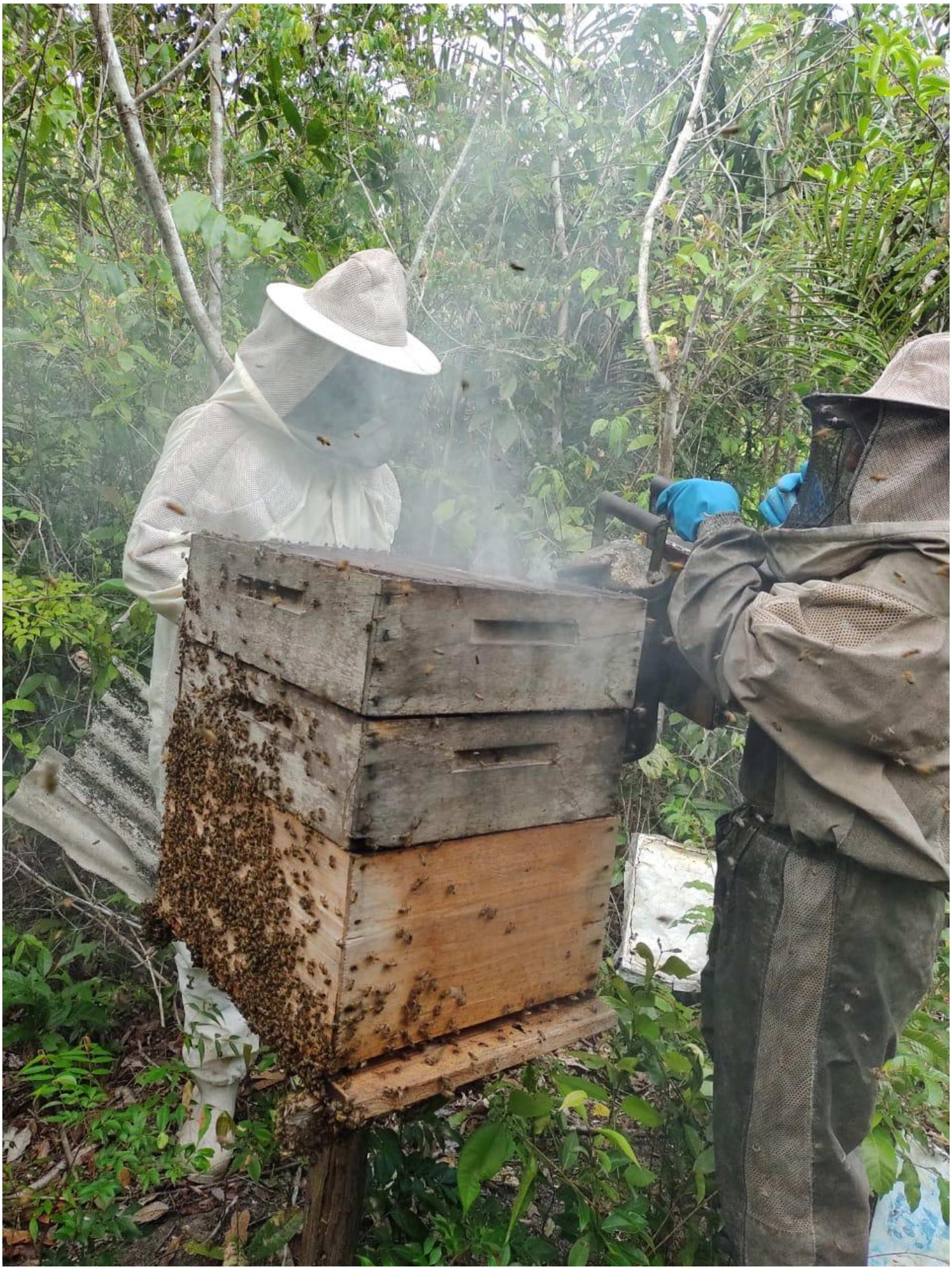

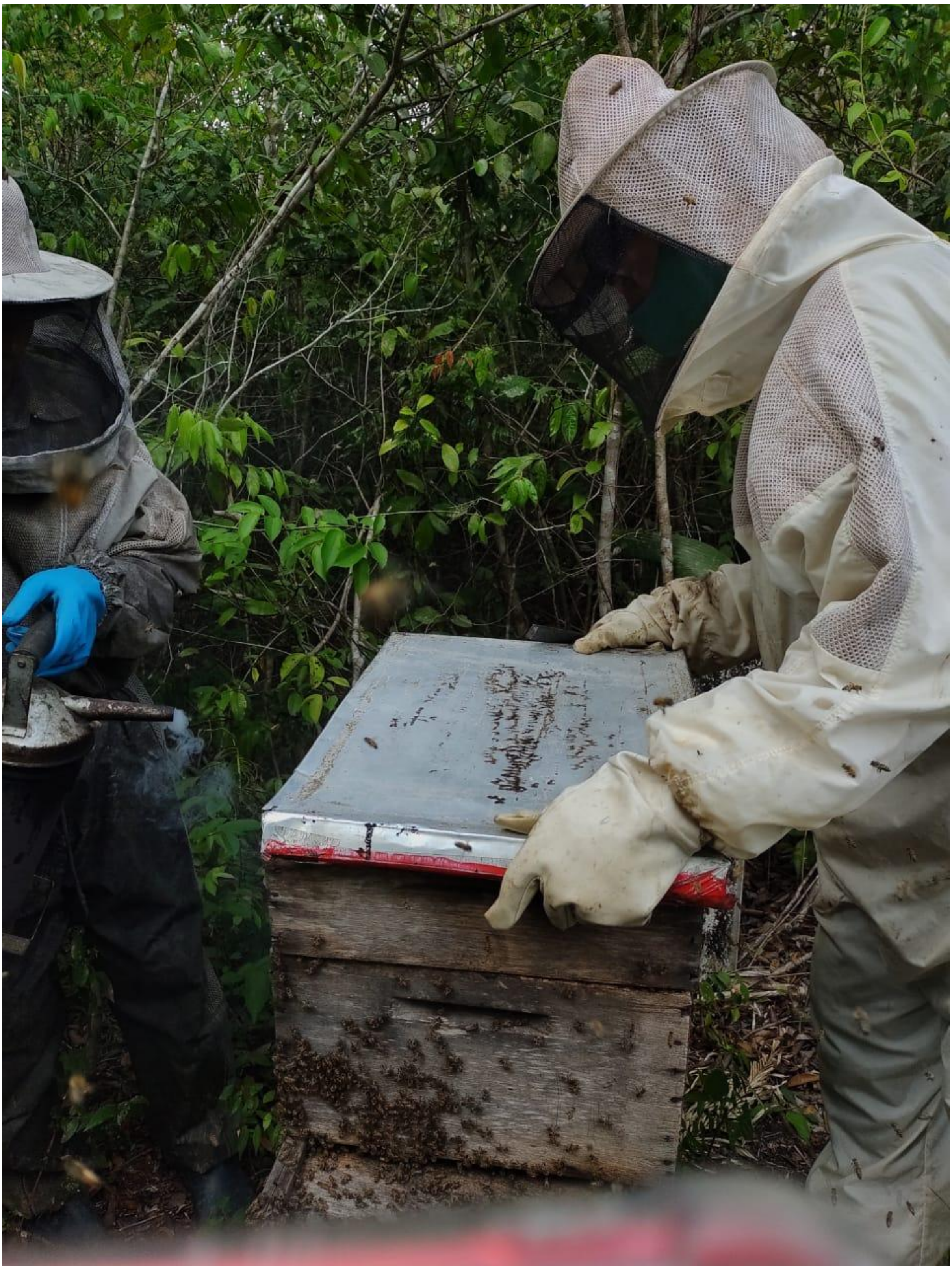

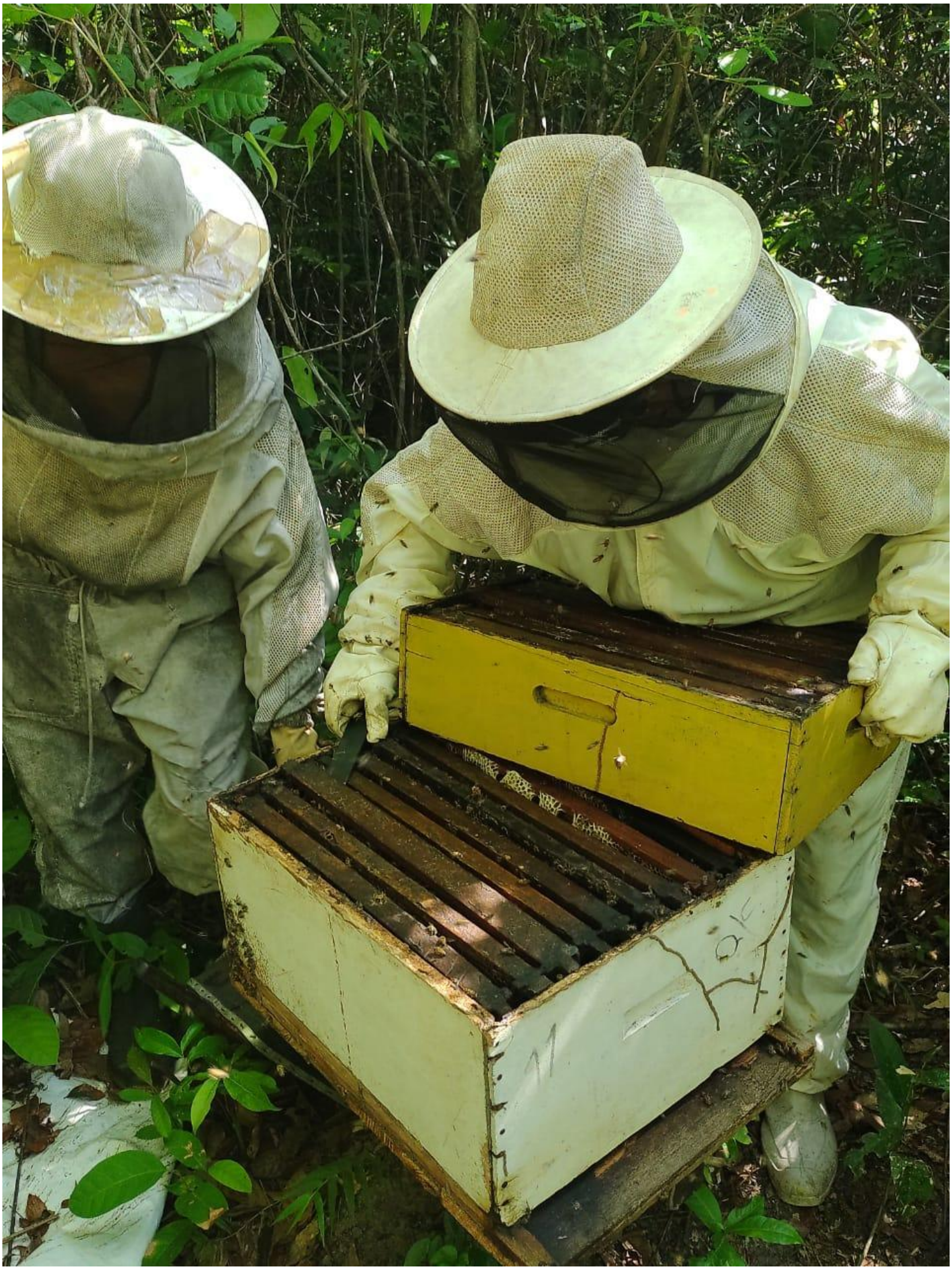

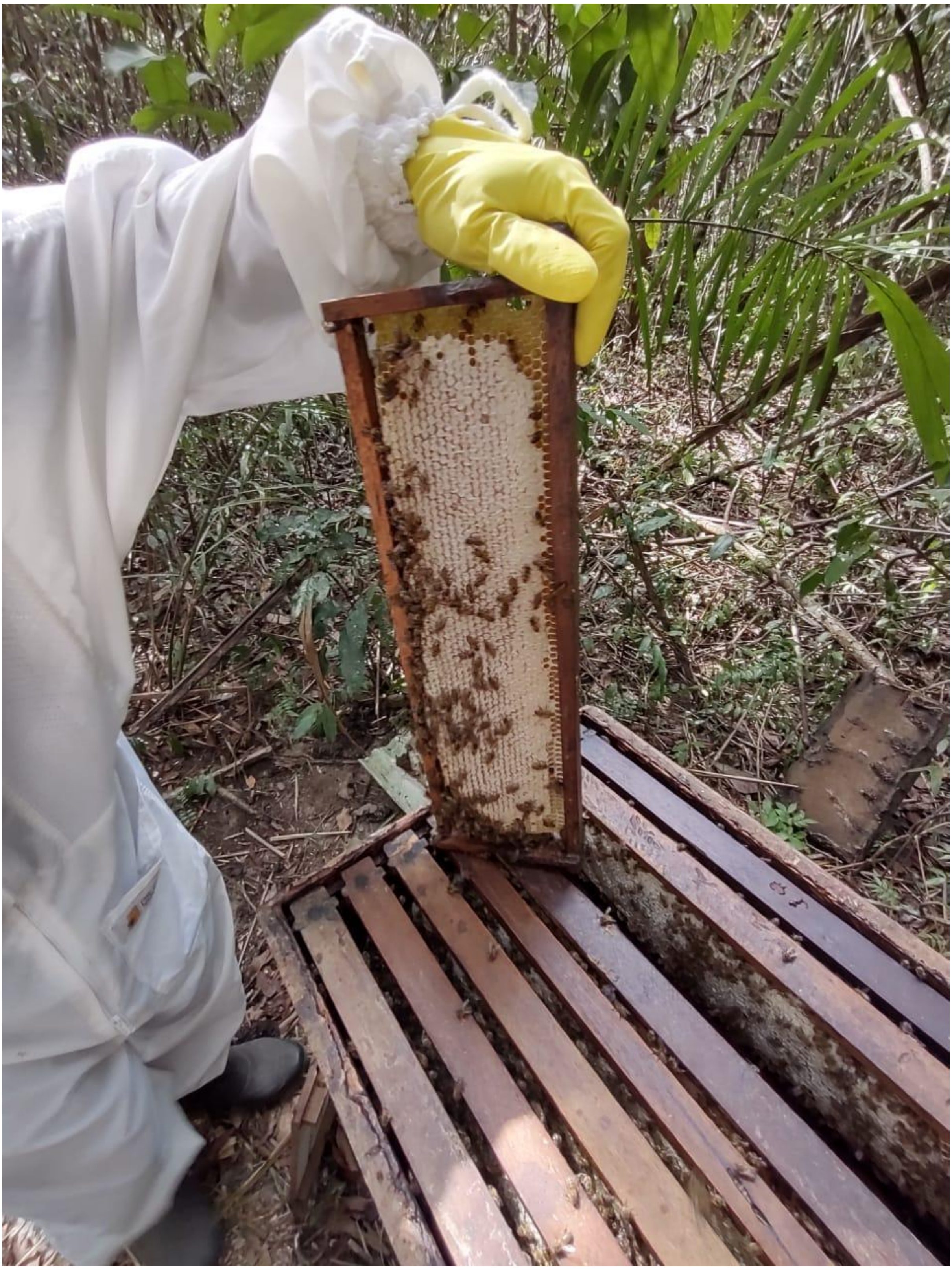

Supplement: Supplementary file 1 [file molecules-26-03462-s001.zip › molecules-1202220-supplementary.pdf]
